# Ecological Differentiation in Two Major Freshwater Bacterial Taxa Along Environmental Gradients

**DOI:** 10.3389/fmicb.2020.00154

**Published:** 2020-02-13

**Authors:** Julia K. Nuy, Matthias Hoetzinger, Martin W. Hahn, Daniela Beisser, Jens Boenigk

**Affiliations:** ^1^Department of Biodiversity, University of Duisburg Essen, Essen, Germany; ^2^Department of Biology and Environmental Science, Linnaeus University, Kalmar, Sweden; ^3^Research Department for Limnology, University of Innsbruck, Mondsee, Austria

**Keywords:** freshwater bacteria, *Polynucleobacter*, *Limnohabitans*, biogeography, ecological differentiation, physicochemical characteristics, microbial eukaryotes, 16S

## Abstract

*Polynucleobacter* (*Burkholderiaceae*, *Betaproteobacteria*) and *Limnohabitans* (*Comamonadaceae*, *Betaproteobacteria*) are abundant freshwater bacteria comprising large genetic and taxonomic diversities, with species adapted to physico-chemically distinct types of freshwater systems. The relative importance of environmental drivers, i.e., physico-chemistry, presence of microeukaryotes and geographic position for the diversity and prevalence has not been investigated for both taxa before. Here, we present the first pan-European study on this topic, comprising 255 freshwater lakes. We investigated *Limnohabitans* and *Polynucleobacter* using an amplicon sequencing approach of partial 16S rRNA genes along environmental gradients. We show that physico-chemical factors had the greatest impact on both genera. Analyses on environmental gradients revealed an exceptionally broad ecological spectrum of operational taxonomic units (OTUs). Despite the coarse resolution of the genetic marker, we found OTUs with contrasting environmental preferences within *Polynucleobacter* and *Limnohabitans* subclusters. Such an ecological differentiation has been characterized for PnecC and LimC before but was so far unknown for less well studied subclusters such as PnecA and PnecB. Richness and abundance of OTUs are geographically clustered, suggesting that geographic diversity patterns are attributable to region-specific physico-chemical characteristics (e.g., pH and temperature) rather than latitudinal gradients or lake sizes.

## Introduction

The extent to which environmental factors constrain the distribution of bacterial freshwater taxa and promote their diversity is a critical subject in microbial ecology. For instance, ubiquitously distributed genera such as *Polynucleobacter* (*Burkholderiaceae*, *Betaproteobacteria*) and *Limnohabitans* (*Comamonadaceae*, *Betaproteobacteria*) comprise a broad diversity of species differentially adapted to physico-chemical parameters including pH and climate conditions (Kasalický et al., 2013; Hahn et al., 2015, 2016; Jezberová et al., 2017). The species diversity harbored by each of these two genera can phylogenetically be clustered into groups characterized by differences in their 16S rRNA genes (Hahn, 2003; Jezbera et al., 2011; Newton et al., 2011; Kasalický et al., 2013). The distribution of such genetic clusters is a function of (I) abiotic (physico-chemical), (II) biotic (e.g., co-occurrence with microeukaryotes) and (III) geographic factors, which may reflect their ecological adaptations, and eventually their potential for dispersal. While *Polynucleobacter* and *Limnohabitans* belong to the best-studied genera in freshwater ecosystems, the relative importance of each of the three factors for their prevalence and diversity has not been investigated in a single study so far.

Both, *Polynucleobacter* and *Limnohabitans*, are abundant in various freshwater ecosystems with diverse environmental conditions (Jezberová et al., 2010; Simek et al., 2010; Newton et al., 2011; Jezbera et al., 2012). For *Polynucleobacter*, five subclusters were described, namely PnecA, PnecB1, and PnecB2 (so far only investigated as PnecB in cultivation-independent studies), PnecC and PnecD (Hahn, 2003; Wu and Hahn, 2006a). PnecC is the best-studied subcluster so far. It comprises multiple described and presumably a large number of undescribed species with diverse ecophysiological and genomic traits, which contrasts their relatively low 16S rRNA gene sequence diversity (Hahn et al., 2016). On the contrary, PnecA is the least investigated subcluster and considered a rare taxon (Hahn et al., 2011). The *Limnohabitans* genus was as well divided into five subclusters LimA, LimB, LimC, LimD, and LimE (Kasalický et al., 2013). Ample knowledge on the ecology of *Limnohabitans* is based on studies employing the R-BT065 FISH probe, which targets bacteria affiliated to four of the subclusters (LimB, LimC, LimD, and LimE) (Simek et al., 2001; Jezbera et al., 2013).

In general, abiotic factors are considered to have the most important impact on bacterial communities (Crump et al., 2007). Several studies addressed the effects of chemical ecosystem features on the distribution of *Polynucleobacter* and *Limnohabitans* subgroups (Jezberová et al., 2010; Simek et al., 2010; Newton et al., 2011; Jezbera et al., 2013). The pH that is often the composition-controlling factor for bacteria in ecosystems (Lindström et al., 2005; Fierer and Jackson, 2006; Logue and Lindström, 2008) was observed to have a strong effect on the distribution of *Polynucleobacter* (Jezberová et al., 2010; Jezbera et al., 2011) and *Limnohabitans* (Simek et al., 2010, 2010; Jezbera et al., 2012). Other distribution limiting factors are hardness of water (conductivity) (Crump et al., 2007), which is often covarying with pH, temperature, and the concentration of dissolved organic carbon (DOC). The DOC composition is strongly coupled to the biological properties of lake ecosystems, as it summarizes external (e.g., humic substances from degraded plant material) and internal (e.g., primary production of phytoplankton) carbon substrate supplies (Moran and Hodson, 1990).

Among biotic factors, protists may shape bacterial communities through grazing (Grujcic et al., 2018) and exudation of organic matter (Horňák et al., 2017). Specifically, culture-based experiments demonstrated that *Polynucleobacter* and *Limnohabitans* strains (LimC, PnecC, and LimB) were preyed on preferentially by flagellates (Šimek et al., 2013; Grujcic et al., 2018). *Limnohabitans* strains from the R-BT cluster exhibited overall higher growth rates in grazing pressure experiments (Simek et al., 2006). Some *Limnohabitans* strains use algal derived organic material as substrate source and show specific substrate utilization characteristics in batch cultures (Simek et al., 2010; Kasalický et al., 2013). In growth experiments on organic matter derived from three axenic algae (genera *Rhodomonas*, *Chlamydomonas*, and *Coelastrum*), LimC strains grew on all sources, while a PnecB strain only grew on organic matter derived from *Coelastrum* (Horňák et al., 2017).

Biogeographical hypotheses have a long history in science and were tested on bacterioplankton community composition and their taxon richness with contradictory results (Horner-Devine et al., 2004; Schiaffino et al., 2013). Hypotheses such as the latitudinal diversity gradient and the species-area relationship postulate a higher richness of taxa with decreasing latitudinal grade and increasing habitat area, respectively. Studies focusing on biogeographic patterns of *Polynucleobacter* and *Limnohabitans* are rare, and most knowledge on this topic is available on the PnecC subcluster (Jezberová et al., 2010; Hahn et al., 2015). To our knowledge, except for PnecC the diversity of *Polynucleobacter* and *Limnohabitans* in relation to geography has not been studied before.

Here, we analyze the distribution and diversity of *Limnohabitans* and *Polynucleobacter* in a large geographical dataset comprising 255 lakes by using amplicon sequencing targeting the V2–V3 region of the 16S rRNA gene for taxonomic assignment. We followed a unique approach to analyze the effects of physico-chemical and geographic lake ecosystem properties, and the co-occurrence with microeukaryotes on the abundance of operational taxonomic units (OTUs) assigned to subclusters. One goal of the study was to assess the power of environmental variables to explain relative abundances of OTUs using variation partition. Further, we correlated relative abundances and diversity measures with environmental gradients to reveal so far uninvestigated ecological trends.

## Experimental Procedures

### Sampling

Sampling of 255 European freshwater lakes was conducted in August 2012, covering a broad latitudinal gradient ranging from Spain to the South of Scandinavia (Supplementary Table S1). The samples were taken by daylight from the shore of each lake or pond collecting epilimnial water up to 0.5 m depth (Supplementary Table S1). Samples were collected under a gradient setting, therefore, the lakes were sampled once and not in replicates. Samples for genomic DNA extraction were filtered onto 0.2 μm nucleopore filters. To obtain similar biomass per sampling site, water was filtered until the filters were blocked by biomass. Biomass filters were subsequently air dried and preserved below −80°C in a cryoshipper (Chart/MVE, Ball Ground, United States) to avoid DNA degradation.

### Assessment of Physico-Chemical and Geographic Factors

Temperature, pH, and conductivity were determined directly in the field in triplicates by use of a Waterproof Tester “Combo” (Hanna Instruments, Vöhringen, Germany). To measure the surface area of each water body, we collected satellite images with corresponding scales from the sampling sites using Google Mymaps (Map data 2017 GeoBasis-DE/BKG (2009), Google). Area calculation of each lake or pond was conducted using ImageJ (v1.8.172; Schneider et al., 2012). The scales of the sampling site images were manually set and an automatic threshold from the 8-bit versions of the images was selected. The particle analysis tool was used to calculate the area in km^2^ of the thresholded images. Coordinates of the sampling sites were obtained with a GPS device.

### DNA Extraction, PCR, and Sequencing

Genomic DNA was extracted from the biomass filters using the my-Budget DNA Mini Kit (Bio-Budget Technologies GmbH, Krefeld, Germany) following the protocol of the manufacturer with minor adaptations. We changed the protocol as follows: Filters were homogenized in 800 μl Lysis Buffer TLS within lysing Matrix E tubes (MP Biomedicals, Santa Ana, CA, United States) and homogenized three times for 45 s using FastPrep (MP Biomedicals, Santa Ana, CA, United States) at 6 m/s followed by incubation for 15 min at 55°C. The quality of the DNA was checked using a NanoDrop^TM^ ND-2000 UV–vis spectrophotometer (Thermo Fisher Scientific, Waltham, MA, United States).

PCR amplifications targeted the V2-V3 region of the 16S rRNA gene using the primers 104F (5′-GGC GVA CGG GTG MGT AA-3′) and 515R (5′-TTA CCG CGG CKG CTG GCA C-3′) (Lange et al., 2015). The selected forward primer contains two wobble positions to catch a broader spectrum of taxonomic groups. Primers with wobble positions containing guanine (G) and cytosine (C) clearly prefer the primer-template binding of *Alphaproteobacteria* DNA, while *Betaproteobacteria* are favored having adenosine (A) in the primer sequence. The binding strength of G-C pairs is higher than that of A-T pairs. Sequences of other taxa that match the primer variants with G or C at the wobble positions, that are *Alphaproteobacteria*, are therefore preferentially bound (Supplementary Table S2). Comparisons within as well as between *Polynucleobacter* and *Limnohabitans* are assumed to be valid, as all known sequences of isolated bacteria affiliated to these taxa exhibit identical sequences at the primer binding sites.

Each sample was amplified twice using primers with different sample identifiers following the AmpliconDuo protocol (A and B variant) (Lange et al., 2015). For the PCR reaction 1 μl of DNA template in 25 μl PCR reaction with 0.4 units of Phusion DNA polymerase (Thermo Fisher Scientific, Waltham, MA, United States), 0.25 μM primers, 0.4 mM dNTPs and 1 × Phusion buffer (Thermo Fisher Scientific, Waltham, MA, United States) were used. The PCR protocol consisted of 35 cycles, including a denaturation step at 98°C for 30 s, annealing step at 72°C for 45 s, and an elongation step at 72°C for 30 s. Finally, the PCR was completed by a final extension step at 72°C for 10 min. Samples were pooled in equimolar ratios and commercially sequenced using paired-end (2 × 300 bp) HiSeq 2500 Illumina sequencing in “rapid-run” mode (Fasteris, Geneva, Switzerland). Eukaryotic data for the same sites were processed as described in Boenigk et al. (2018).

### Bioinformatic Analyses

We used the standardized workflow Natrix^1^ to analyze the amplicon data. Adapter removal, quality trimming, and demultiplexing using indexed sequences was performed by the sequencing company (Fasteris, Geneva, Switzerland). Thereupon, base quality of raw sequence reads was checked using the FastQC software (v0.11.8, Andrews, 2018). The raw sequences were quality filtered to remove reads with an average Phred quality score below 25 using PRINSEQ-lite (v0.20.4; Schmieder and Edwards, 2011). Additionally, all reads with at least one base with a Phred quality score below 15 were removed. The paired-end reads were assembled and quality filtered with the tool PANDASeq (v2.10; Masella et al., 2012). The remaining reads were dereplicated and chimeras were removed using UCHIME with default parameters (usearch v7.0.1090; Edgar et al., 2011). Finally, a split-sample filtering protocol for Illumina amplicon sequencing (AmpliconDuo) was used as described in Lange et al. (2015) for the removal of sequence artifacts. Sequences that were not found in both sample branches (A and B variant) were discarded resulting in 128,766,060 reads. Filtered reads were clustered in OTUs via the software SWARM using default settings (v2.2.2; Mahé et al., 2014) resulting in 145 759 OTUs. Taxonomic assignment was performed using the database SILVA (SILVA SSURef release 132). *Betaproteobacteriales* were reassigned to the class *Betaproteobacteria*.

**FIGURE 1:**
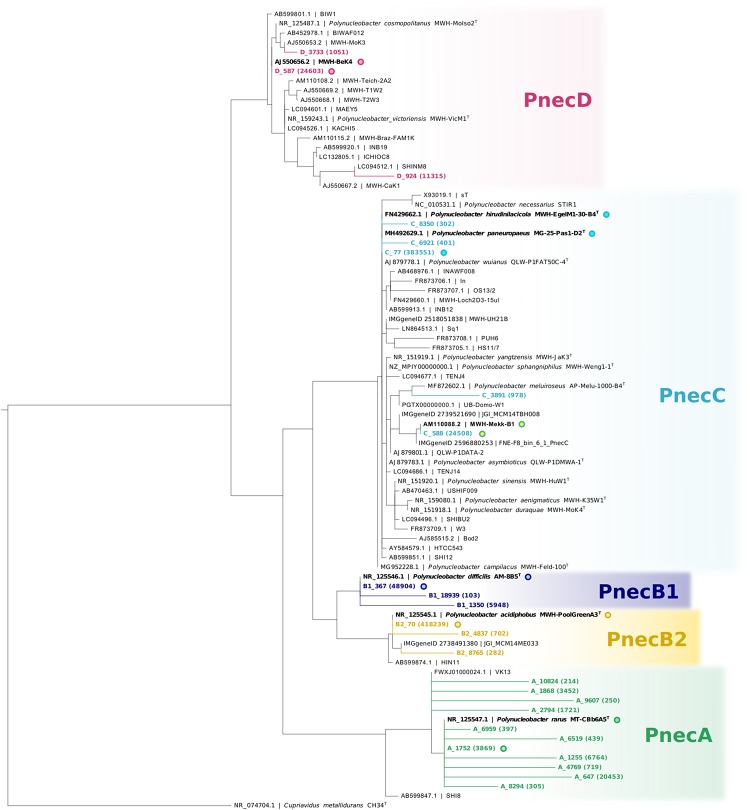
Phylogeny of *Polynucleobacter* OTUs with respect to 16S rRNA gene sequences of isolated strains. The centroid sequences (Mahé et al., 2014) representing all *Polynucleobacter* OTUs used in this study (in colors) were integrated into a RAxML backbone tree by phylogenetic placement. The backbone tree is based on the alignment of 16S rRNA gene sequences of isolated strains. Redundant reference sequences were excluded, except for two identical sequences within PnecC that represent different species. Designations of reference sequences consist of accession IDs before the vertical bar, and strain names after the vertical bar. Sequences are available either in GenBank or the IMG/ER database. Accession IDs referring to the latter database are designated accordingly. Sequence identities between OTUs and sequences of the backbone tree are highlighted by circles with common fill color. Numbers in brackets show the read count of each OTU in the whole dataset.

**FIGURE 2:**
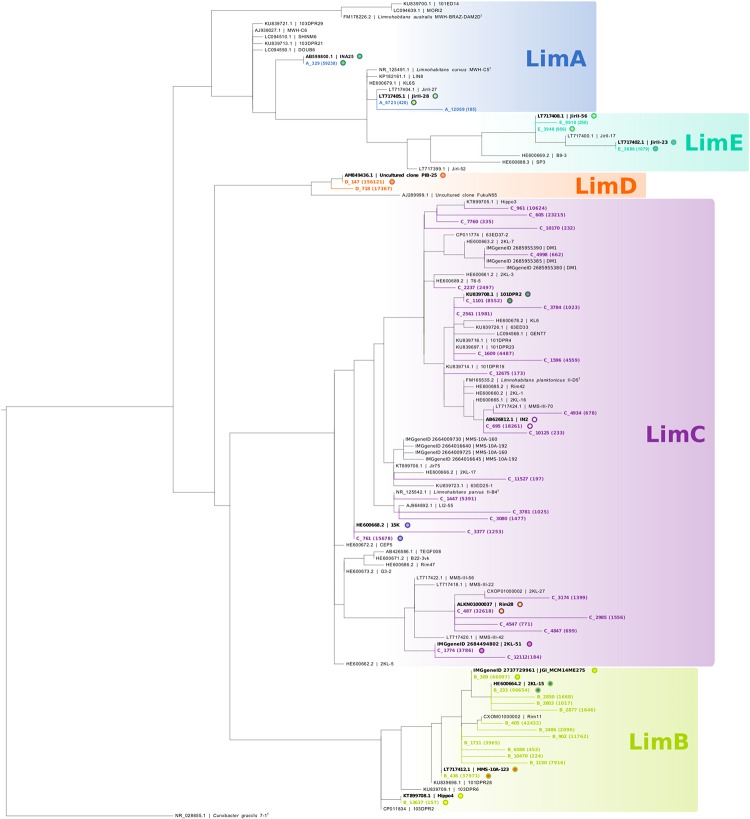
Phylogeny of *Limnohabitans* OTUs with respect to 16S rRNA gene sequences of isolated strains. The tree was generated analogous to Figure 1, i.e., all *Limnohabitans* OTUs (in colors) were used for phylogenetic placement, and the backbone tree is based on 16S rRNA gene sequences of isolated strains. Two sequences from uncultured clones of LimD were allowed in the backbone tree, as no cultured representatives of this subcluster are publicly available. Some *Limnohabitans* strains comprise multiple ribosomal operons, and are represented by multiple sequences in case of sequence differences among different 16S rRNA gene copies.

As we focus in particular on *Polynucleobacter* (1,076,191 reads; 852 OTUs) and *Limnohabitans* lineages (729,321 reads; 1 230 OTUs), we assigned the respective OTUs to subclusters of these genera (PnecA, PnecB1, PnecB2, PnecC, and PnecD within *Polynucleobacter* and LimA, LimB, LimC, LimD, and LimE within *Limnohabitans*). In course of this assignment, specific chimera checks were applied to exclude chimeric sequences that had not been removed by UCHIME. To do so, the sequences were split in front and rear parts [front sequence (258 bp) and rear sequence (247 bp)] and blasted independently against the SILVA database. Sequences for which the taxonomic assignment of front and rear part did not match at the genus level were removed. Kept sequences were subjected to a further chimera check on the subcluster level. Front and rear sequences were independently assigned to the subclusters by phylogenetic placement (Janssen et al., 2018), and those sequences for which the assignment did not match were removed. The trees underlying the phylogenetic placement have been calculated as follows. 16S rRNA gene sequences of isolated *Polynucleobacter* and *Limnohabitans* strains were collected from GenBank (Clark et al., 2016). Solely two uncultured clones representing LimD were included, as no sequences of cultured representatives of this subcluster are publicly available. As outgroups, sequences of *Cupriavidus metallidurans* strain CH34^T^ and *Curvibacter gracilis* strain 7-1^T^ were included for *Polynucleobacter* and *Limnohabitans*, respectively. Sequences were aligned with muscle (Edgar, 2004) using the MEGA X software (Kumar et al., 2018). Alignments were trimmed to a length of 1384 bp for *Polynucleobacter* and 1322 bp for *Limnohabitans*. Shorter sequences as well as identical sequences were excluded from the alignment. Phylogenetic trees were calculated from these alignments using RAxML with the rapid hill-climbing algorithm and the GTRGAMMA nucleotide substitution model (Stamatakis, 2015). The trees including the phylogenetic placement of those OTUs that were finally kept are shown in Figures 1, 2. The applied filter method resulted in 25 *Polynucleobacter* OTUs accounting for 950,890 reads and 51 OTUs assigned to *Limnohabitans* subclusters with 631,159 reads. All OTUs and the respective number of reads are given in Supplementary Table S3. We have refrained from assigning OTUs on species level, as species within the selected genera cannot be resolved based on 16S rRNA gene sequences (Kasalický et al., 2013; Hahn et al., 2016). This is also evident in Figure 1 depicting two different PnecC species with identical sequences, which are thus represented by the same OTU (C_77).

### Statistical Analyses

Prior to statistical analyses conducted using R v3.5.2 (R Core Team, 2018), each dataset was filtered for unreliable OTUs. To detect these OTUs, the samples were normalized by the total number of assigned reads per sample and each OTU in a sample was discarded with a relative abundance below a global threshold of 0.01%. The relative read abundances of *Limnohabitans* and *Polynucleobacter* OTUs among all bacteria were used to investigate their occurrence and frequency in presence of different environmental factors (temperature, pH, lake surface area, conductivity, and geographical position) visualized in jittered scatter plots (ggplot2 package, v3.1.0; Wickham, 2011). To explain the variation in the distribution of *Polynucleobacter* and *Limnohabitans* subclusters by (1) physico-chemical factors, (2) biotic factors (protistan community) and (3) geography on the distribution of *Polynucleobacter* and *Limnohabitans* subcluster, we applied variation partitioning (Blanchet et al., 2008). The response matrices are dissimilarity matrices (Bray–Curtis) of bacterial Hellinger-transformed community data (on basis of OTUs) of *Polynucleobacter* or *Limnohabitans*. The physico-chemical, biotic or geographical dataset was used accordingly as explanatory variable. The environmental dataset included five chemical and topographical parameters (Supplementary Table S1). The biotic dataset consisted of community data of microeukaryotes. The geographical dataset comprised Moran Eigenvector maps (MEMs) created by function dbmem, cf, distances ≤852 km (Blanchet et al., 2008), geographical latitude and longitude. MEMs decompose the spatial structure of a dataset into a large number of Eigenfunctions.

To investigate correlations of abiotic factors and the relative abundance of *Polynucleobacter* and *Limnohabitans* subclusters, Spearman rank correlations were calculated and visualized with the corrplot package v0.84 (Wei and Simko, 2017). To investigate relations between eukaryotic taxa and the subclusters of *Polynucleobacter* and *Limnohabitans* without the influence of covarying abiotic factors, we calculated Kendall partial rank correlations. Fungi, Metazoa, and mainly parasitic taxonomic groups were excluded from the eukaryotic dataset. Correlations with *p*-values below 0.05 were considered as significant. The correlations were visualized in a co-occurrence network using Cytoscape v.3.7.1 (Shannon et al., 2003). Correlation coefficients were represented as edges, and subcluster and eukaryotes were shown as nodes.

Further, we calculated diversity indices, i.e., OTU richness, Shannon index, Simpson index, and Evenness to analyze the effect of geography on diversity. We plotted the OTU richness and other diversity indices per sample and pie-charts depicting the subcluster composition on a map of Europe. To relate environmental factors to patterns of taxon richness and diversity indices, pH, area, and temperature of each sample were visualized with color gradients. The maps were generated with the rworldmap package v1.3-6 (South, 2011).

### Data Availability

Raw amplicon sequences from the prokaryotic samples were deposited in the Sequence Read Archive at NCBI under Bioproject accession number PRJNA559862. Eukaryotic samples are available under Bioproject PRJNA414052.

## Results

### Influence of Environmental Factors

To analyze the influence of the environmental factors, i.e., geography, microeukaryotes and physico-chemistry on *Polynucleobacter* and *Limnohabitans*, we conducted variation partition analyses. The considered environmental factors explained overall 32.90% of the total variation of the *Polynucleobacter* community, while the same factors explained overall 15.70% of the total variation of the *Limnohabitans* community (Table 1). Of the total explained variation of *Polynucleobacter*, physico-chemical variables (pH, temperature, area, conductivity, and elevation) accounted for approximately 50%. The intersection of physico-chemistry and geography (latitude, longitude and MEMs) explained together 16.77% while geography alone explained 10.37%. The community composition of microeukaryotes explained with 3.66% a minor part of the variation. For *Limnohabitans*, physico-chemistry and the intersection of physico-chemistry and geography accounted for 26.32% each of the total variation. Geography and the community composition of microeukaryotes explained with 18.66 and 11.96%, respectively, a higher extent of the variation in *Limnohabitans* in comparison to *Polynucleobacter*.

**TABLE 1:** Relative contribution of environmental variables, explaining the variation of *Polynucleobacter* and *Limnohabitans*.

Variation	Explanatory variables	*Polynucleobacter* (%)	*Limnohabitans* (%)
Total explained variation		32.9	15.7
	Geo	10.37	18.66
	Euks	3.66	11.96
	Physchem	49.70	26.32
	Geo + Physchem	16.77	26.32
	Geo + Euks	3.05	6.22
	Physchem + Euks	9.45	7.18
	Geo + Euks + Physchem	7.01	3.35
Unexplained variation		67.1	84.3

*Geography (Geo) comprises the latitudinal and longitudinal position of the lakes and computed dbMEMs. Physico-chemistry (Physchem) comprises elevation, lake surface area, pH, temperature, and conductivity. The eukaryotic community (Euks) consists of all OTUs assigned to microbial eukaryotes.*

### Taxonomic Community Composition

*Bacteroidetes*, *Alphaproteobacteria*, and *Cyanobacteria* represented, despite site-specific variations, the major bacterial groups detected across the sampled lakes (38 ± 25, 19 ± 15, and 27 ± 22% of total bacterial reads). *Betaproteobacteria* contributed 3 ± 5% of all bacterial reads. Reads affiliated to *Polynucleobacter* accounted for 39 ± 55% and reads affiliated to *Limnohabitans* accounted for 19 ± 27% of all reads assigned to *Betaproteobacteria* (Table 2 and Supplementary Figure S1).

**TABLE 2:** Relative composition of the bacterial community.

Bacterial taxa		Mean (%)	SD (%)
Acidobacteria		0.04	0.11
Actinobacteria		**4.12**	**4.68**
Alphaproteobacteria		**19.66**	**15.22**
Armatimonadetes		0.00	0.03
Bacteroidetes		**37.50**	**24.62**
Betaproteobacteria		**3.25**	**4.58**
*Limnohabitans*		0.61 (18.67)	1.22 (26.77)
*Polynucleobacter*		1.27 (39.09)	2.53 (55.25)
Chloroflexi		0.17	0.30
Cyanobacteria		**26.66**	**22.14**
Deinococcus		0.19	0.48
Deltaproteobacteria		0.55	0.89
Fibrobacteres		0.00	0.01
Firmicutes		**0.20**	**0.55**
Fusobacteria		0.01	0.02
Gammaproteobacteria		**0.89**	**1.70**
Gemmatimonadetes		0.36	0.93
Nitrospinae		0.00	0.01
Planctomycetes		**0.01**	**0.06**
Spirochaetes		0.01	0.02
Synergistetes		0.00	0.01
Tenericutes		0.00	0.01
Verrucomicrobia		**6.37**	**11.44**

**Subcluster**	**No. OTUs**	**Mean (%)**	**SD (%)**

***Limnohabitans***			
LimA	3	5.76	15.86
LimB	14	47.06	30.80
LimC	28	22.91	30.58
LimD	2	23.56	27.35
LimE	3	0.71	6.74
***Polynucleobacter***			
PnecA	11	3.96	8.24
PnecB1	3	6.65	12.83
PnecB2	3	53.06	41.66
PnecC	4	31.02	38.72
PnecD	3	5.31	15.18

*Mean and standard deviation of the relative abundances of bacterial taxa across all samples were calculated. The relative subcluster composition of *Limnohabitans* and *Polynucleobacter* is separately shown. Numbers in brackets refer to the proportion of *Limnohabitans* and *Polynucleobacter* within the class *Betaproteobacteria*. The most abundant phyla and classes are highlighted with bold font.*

The bacterial dataset showed an unusual composition of the relative abundances of taxa in comparison to various other freshwater lake studies. Considering the major proteobacterial classes, *Betaproteobacteria* seem to be underrepresented, while *Alphaproteobacteria* might be overrepresented (Table 2). OTUs of the *Limnohabitans* genus were assigned to five subclusters. LimA (3 OTUs), LimB (14 OTUs), LimC (28 OTUs), LimD (2 OTUs), and LimE (3 OTUs). Subclusters LimB, LimD, and LimC were most abundant (47 ± 31, 24 ± 27, and 23 ± 31%, respectively), while subclusters LimA and LimE represented the minor fraction of the *Limnohabitans* genus across samples (Table 2). Yet, LimA comprised the second most abundant *Limnohabitans* OTU (A_329, 59,238 reads), which shares 100% sequence identity with strain INA25 (Figure 2). The most abundant OTU was D_147 with 156,121 reads, sharing sequence identity with the uncultured clone PIB-25 (Figure 2).

Operational taxonomic units of the *Polynucleobacter* genus were assigned to five subclusters. PnecA (11 OTUs), PnecB1 (3 OTUs), PnecB2 (3 OTUs), PnecC (4 OTUs), and PnecD (3 OTUs). PnecB2 (53 ± 42%), and PnecC (31 ± 39%) represented the major fraction within the *Polynucleobacter* genus across samples. Although PnecA contained the most OTUs, the PnecA subcluster had a comparably low relative abundance (4 ± 8%). Also the PnecD subcluster comprised a small relative fraction of reads (5 ± 15%). The most abundant *Polynucleobacter* OTUs were B2_70 with 418 239 reads (sequence identity with the type strain of *P. acidiphobus*) and C_77 with 383 551 reads (sequence identity with the type strains of *P. paneuropaeus* and *P. hirudinilacicola*) (Figure 1).

### Relations of *Polynucleobacter* and *Limnohabitans*

Relative abundances of *Polynucleobacter* and *Limnohabitans* genera were not significantly related to each other along environmental gradients (Supplementary Figure S2). On subcluster level several subclusters were strongly correlated with one another (Supplementary Figure S3). In this respect, PnecA positively covaried with PnecC, LimB, and LimD. Both, PnecC and LimB had high correlation coefficients with LimD. PnecB2 had the highest negative correlation coefficient with PnecC.

Supplementary Figure S4 shows networks of correlations of OTUs based on relative read abundances. The network of positive correlations roughly separated into two pole-like clusters with strongly interconnected groups of OTUs (Supplementary Figure S4a). The left cluster is dominated by OTUs that are negatively correlated with pH, while the OTUs within the right cluster were predominantly associated with higher pH (Supplementary Figure S5). The two most abundant *Polynucleobacter* OTUs C_77 and B2_70 are located within the left and right cluster, respectively, and were strongly negatively correlated with each other (Supplementary Figure S4b). The OTU C_77 positively covaried with two of the most abundant *Limnohabitans* OTUs B_405 and D_147, while B2_70 was positively correlated with the LimB OTUs B_233, B_309 and B_436.

### Composition in Dependency of Physico-Chemical Factors

**FIGURE 3:**
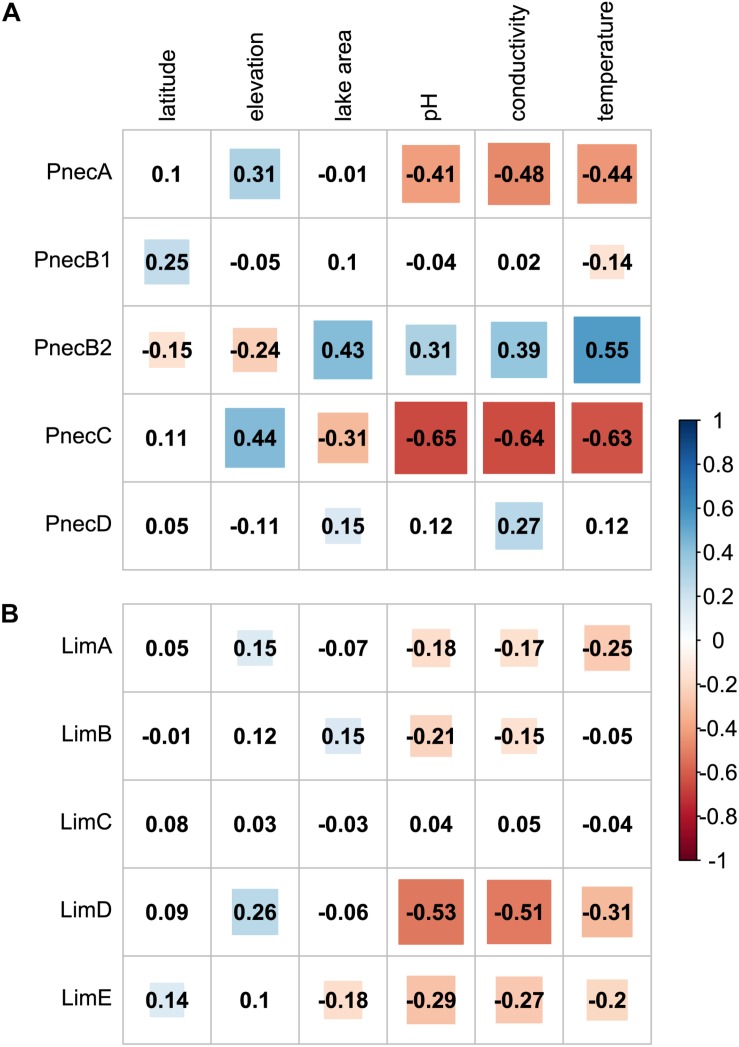
Spearman rank correlations of subcluster abundances with environmental parameters. Correlation coefficients for **(A)** *Polynucleobacter* and **(B)** *Limnohabitans* subclusters are provided for each correlation pair. Significant correlations (*p* < 0.05) are highlighted in blue for positive, and red for negative correlations.

PnecC exhibited the strongest associations with environmental factors (Figure 3 and Supplementary Figure S6). The relative read abundance of PnecC was negatively correlated with pH, conductivity, temperature, and lake surface area. Similarly, PnecA was associated with low pH, conductivity and temperature. Compared to PnecA and PnecC, PnecB2 exhibited inverse trends, i.e., this subcluster was preferentially detected at higher pH, conductivity, water temperature, and lake surface area. PnecB1 only correlated positively with latitude and negatively with temperature, and PnecD covaried positively with conductivity and area. Relations of the *Limnohabitans* subclusters LimA, LimB, LimD, and LimE were overall negative with pH, conductivity and except for LimB with temperature. LimB was positively correlated with area and LimE was negatively associated with area and positively with latitude. LimC exhibited no significant correlations to any tested environmental factor.

**FIGURE 4:**
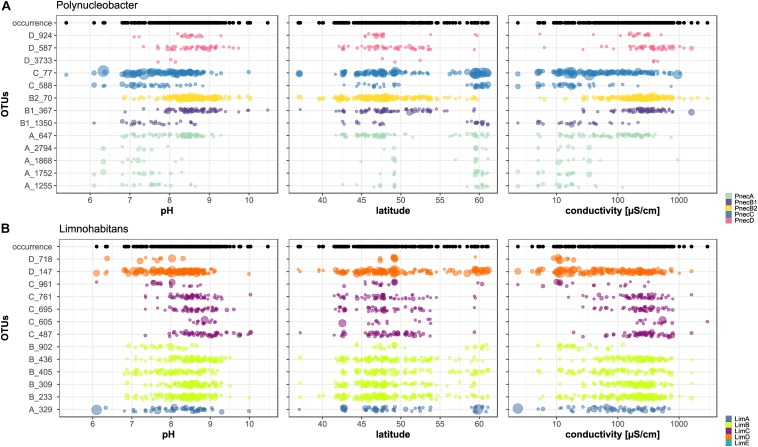
Abundances of OTUs across environmental gradients. Only the 13 most abundant OTUs assigned to **(A)** *Polynucleobacter* and **(B)** *Limnohabitans* are shown. The size of each dot is proportional to the OTU’s relative read abundance in the bacterial community. Black dots above each figure illustrate the occurrence of **(A)** *Polynucleobacter* and **(B)** *Limnohabitans* at the sampling sites.

The correlations of all OTUs with environmental parameters are presented in Supplementary Figure S5, which shows that not all OTUs associated to one subcluster exhibited coherent ecological signals. For example, LimB was negatively correlated with pH and conductivity, while OTU B_436 revealed positive correlations with these parameters. The abundance of *Limnohabitans* and *Polynucleobacter* OTUs as a function of pH, latitude and conductivity is shown in Figure 4. Only the thirteen most abundant OTUs per genus were included. The results for all OTUs are presented in Supplementary Figure S7. For *Polynucleobacter*, OTU C_77 shows the widest occurrence spectrum. It was merely absent in samples with pH > 10 and conductivity >1000 μS cm^–1^. The second PnecC OTU C_588 was detected in a narrower pH range (6–9) and seems to be preferentially abundant at the highest sampled latitudes (Southern Scandinavia). This geographic area, which comprises mostly acidic to circum-neutral lakes, was clearly dominated by the two PnecC OTUs in terms of read abundance.

The overall most abundant OTU B2_70 was the only *Polynucleobacter* OTU detected at the highest sampled conductivities (up to 2917 μS cm^–1^), but was restricted to pH > 6.8. Considering the geographic distribution of this OTU, it is conspicuous that it dominated many lakes in the Alps and its foothills. Generally, alpine lakes seem to be either dominated by OTU B2_70 [e.g., Lac de Annecy (Z163AC), Lac du Chevril (Z121CH), Kloentalersee (A281KL), Walensee (A272WA), Ammersee (A291AM), lake Wallersee (A102WA), lake Weissensee (A131WE), lake Erlaufsee (A042ER)] or C_77 [e.g., O271GA, Altausseer lake (A112AL), Obersee (A123OB), Morskie Oko (O041MO) and Popradske Pleso (O053PO)]. Exceptions are Rothsee (O261RO) and Plansee (A242PL), which contained a high relative abundance of PnecD reads (Supplementary Figure S8).

The two PnecB1 OTUs showed rather contrasting conductivity preferences, with B1_367 detection ranging from 27 to 1599 μS cm^–1^ and B1_1350 from 2 to 214 μS cm^–1^. PnecA and PnecD seem to consist of OTUs with coherent and more restricted habitat ranges than those of other subclusters. PnecA OTUs were preferentially detected at low conductivities, and those of PnecD at high conductivities. Interestingly, both PnecA and PnecD were not detected in Southern Europe (<42° latitude), yet, only 14 samples were analyzed from this region (Supplementary Figure S8).

Several *Limnohabitans* OTUs were not detected along the whole sampled pH gradient but restricted to relatively narrow pH ranges. For instance, no LimC OTU was detected at pH < 7.3, with only one exception (OTU C_961 at pH 6.1). The OTU C_605 revealed an exceptionally restricted pH range (8.4–9.2). Similarly, the distribution of LimB seems to be restricted by pH. All OTUs assigned to LimB showed a similar pH range, i.e., the subcluster was detected in only one sample with pH > 9.5, and not at all at pH < 6.8. On the contrary, LimA and LimD OTUs show a broader range toward low pH values, and were detected at a minimum pH of 6.1. LimD exhibited a high relative read abundance in the samples from Scandinavia. Conversely, LimB and LimC dominated the samples from Central and Eastern Europe (Supplementary Figure S8).

### Operational Taxonomic Unit Diversity

**FIGURE 5:**
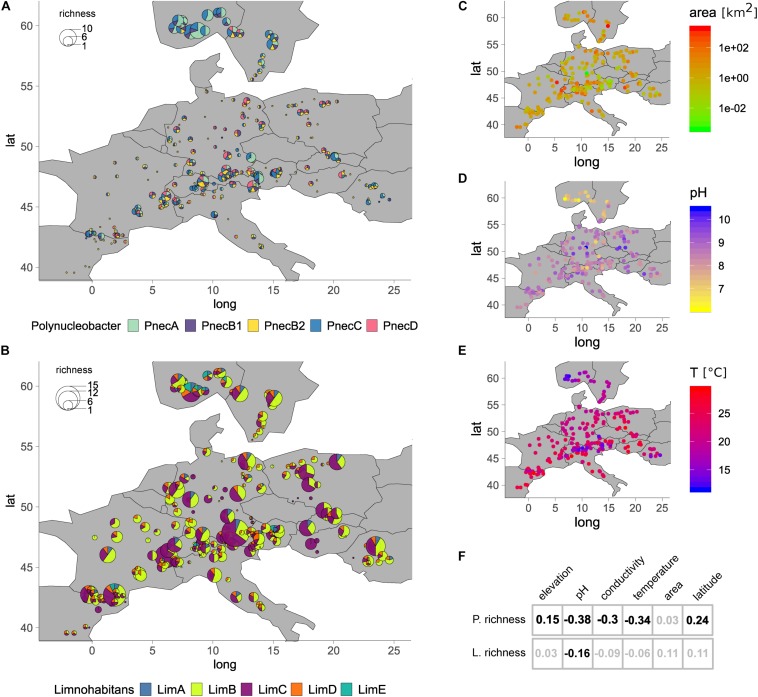
Geographic maps showing the OTU richness of each sample. The OTU richness of **(A)** *Polynucleobacter* and **(B)** *Limnohabitans* is represented by the size of each piechart. Piecharts are divided according to the number of OTUs associated to the different subclusters. The maps in panels **(C–E)** illustrate lake area, pH and temperature for each sample, respectively. The table in panel **(F)** shows Spearman rank correlation coefficients of environmental factors with OTU richness of *Polynucleobacter* and *Limnohabitans*. Significant correlations (*p* < 0.05) are shown in black, not significant correlations in gray font. Sites with higher *Polynucleobacter* OTU richness have lower temperature and acidic to circum-neutral pH.

Operational taxonomic unit richness, Shannon index (H), and Simpson index (SI) revealed overall similar correlations with environmental factors for the investigated samples. The OTU richness, H and SI of *Polynucleobacter* in the investigated samples was positively correlated with latitude and elevation, and negatively with pH, conductivity and temperature (Figure 5 and Supplementary Figure S9). The Evenness was positively correlated with latitude and negatively correlated with elevation and temperature. Surface area of lakes was not significantly correlated with OTU richness, H, SI and Evenness. In the sampled latitudinal gradient, the OTU richness, H and SI did not gradually increase but samples with high diversity were concentrated in two major regions (Figure 5 and Supplementary Figures S9, S10). The highest richness and diversity indices of *Polynucleobacter* communities were found in the northern area between 57° latitude and the northern edge of the entire sampling area at 63°comprising 17 sites. The second area with sites of high OTU richness and H were an alpine area between 45° and 49° latitude containing 11 sites (elevation: 427–1793 m). Exceptions from this pattern were two sampling sites in eastern Europe and two sites in the northern Alps also comprising high richness. The analysis of SI did not show high values in the alpine area. The Evenness of *Polynucleobacter* shows an inverted pattern compared to the richness and the Shannon index. The Evenness is low in the Scandinavian lakes and highest in the alpine region (Figure 5 and Supplementary Figure S10).

The sites in the northern area were characterized by a high number of OTUs affiliated to PnecA. The lakes with highest richness were among the sites with lowest pH (6–7), conductivity and temperature (>20°C). The OTU richness of *Limnohabitans* was only negatively correlated with pH, and SI and H were positively correlated with lake area. The Evenness was positively correlated with pH and conductivity and negatively with elevation. OTU richness and diversity indices did not show an apparent geographical pattern (Figure 5 and Supplementary Figure S10).

### Interactions of Bacteria and Microeukaryotes

**FIGURE 6:**
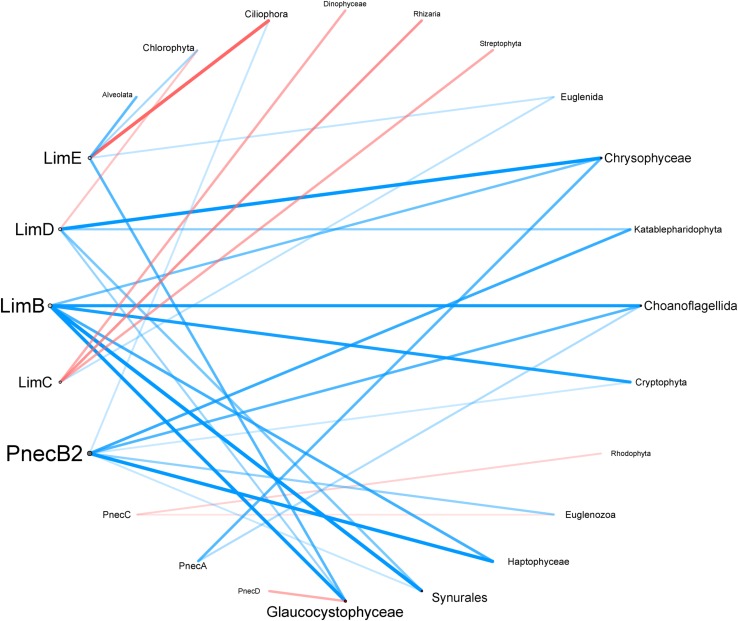
Correlation network of microeukaryotes with *Polynucleobacter* and *Limnohabitans* subclusters. Correlation coefficients are based on partial Kendall correlation analysis and represented by edge thickness. Positive and negative correlations are shown in blue and red, respectively. The number of edges connected to each node is visualized by the size of its name. Only significant correlations (*p* < 0.05) are shown.

The interaction network depicted in Figure 6 shows the partial correlations of eukaryotes with *Polynucleobacter*, and *Limnohabitans* subclusters. Partial correlations were calculated to exclude the co-variation effects of environmental factors (latitude, area, elevation, pH, temperature and conductivity) on protists and both genera. The network consists of eight nodes from *Polynucleobacter* and *Limnohabitans*, and sixteen nodes from eukaryotic groups. Out of 31 edges, 24 indicated a positive correlation, while 7 indicated negative correlations. PnecB2 and LimB have with seven and six connected edges to protists the most connecting edges among all tested subclusters. PnecB2 and Haptophyceae possessed with *r*_*k*_ = 0.18, *p* = 1.47 × 10^–4^ the highest correlation coefficient within the *Polynucleobacter* genus (Supplementary Table S4). As revealed in Supplementary Figure S11, OTU B2_70 exhibits the most connections to protists among B2 OTUs and has the highest correlation coefficient with Haptophyeae [*r*_*k*_ = 0.182, *p* = 1.45 × 10^–4^ (Supplementary Table S5)]. PnecB2 has connecting edges to Choanoflagellida, Cryptophyta, Haptophyta, Synurales, Euglenozoa, Ciliophora, and Katablepharidophyta. LimB and Synurales possessed with *r*_*k*_ = 0.18, *p* = 1.28 × 10^–4^, the highest positive correlation coefficients within the *Limnohabitans* genus (Supplementary Table S4). The OTU network revealed that LimB was correlated with similar microeukaryotes compared to B2_70 with highest correlation coefficients between B_436 and Glaucocystophyceae (*r*_*k*_ = 0.240, *p* = 1.11 × 10^–6^), and B_309 and Haptophyceae (*r*_*k*_ = 0.212, *p* = 1.71 × 10^–5^). In general, LimB is connected through positive correlations with Chrysophyceae, Choanoflagellida, Cryptophyta, Haptophyta, Synurales, and Glaucocystophyceae (Figure 6).

LimC has three connecting edges that show negative correlations to Dinophyceae, Rhizaria, and Streptophyta and one positive correlation to Euglenida. LimC OTUs show overall the strongest negative correlations with protists, in particular with Katablepharidophyta and Haptophyceae (Supplementary Figure S11b and Supplementary Table S5). Further, groups of LimC OTUs positively correlated with similar protists. For example, C_4547, C_4847, C_1774, and C_3784 positively correlated with Ciliophora, and C_605, C_487, C_1609, C_695, C_1101, C_2561, and C_12575 with Euglenida. The strongest positive correlation is visible between Apusozoa and C_2985 with *r*_*k*_ = 0.694, *p* = 614 × 10^–45.^ PnecC and PnecA have two connecting edges each. While PnecA positively correlates with Chrysophyceae and Choanoflagellida, PnecC negatively correlates with Rhodophyta and Euglenozoa.

Among eukaryotes, Glaucocystophyceae (four edges) and Chrysophyceae, Choanoflagellida, and Synurales (three edges) have the most connecting edges to bacterial subclusters. Chlorophyta, Ciliophora, Euglenida, Katablepharidophyta, Cryptophyta, Euglenozoa, and Haptophyta have two connecting edges each.

## Discussion

Here, we investigated for the first time the joint effects of physico-chemical factors, presence of microeukaryotes, and geography on two abundant freshwater taxa, *Polynucleobacter* and *Limnohabitans*. Our analyses clearly indicate physico-chemical factors having the strongest influence on subclusters and OTUs of both taxa (Table 1). In this context, we could corroborate known ecological preferences of subclusters, most notably with respect to pH and conductivity. In accordance with previous studies, abundances of PnecA (Newton et al., 2011), PnecC (Jezberová et al., 2010; Jezbera et al., 2011, 2012; Newton et al., 2011; Watanabe et al., 2012), LimA (LhabA3 in Newton et al., 2011; Shabarova et al., 2017), and LimD (Lhab-A2 in Newton et al., 2011) were negatively correlated with pH (Figure 3 and Supplementary Figure S6). We also found associations with low pH for LimB and LimE, which to our knowledge have not been analyzed as separate groups in previous cultivation-independent studies. Interestingly, most studies employing the R-BT FISH probe, which targets LimB, LimC, LimD, and LimE, reported positive relations with pH (Simek et al., 2010; Jezbera et al., 2012), while we did not find a significant positive correlation for any *Limnnohabitans* subcluster. Among *Polynucleobacter*, the only subcluster preferentially abundant at high pH was PnecB2, which conforms to earlier studies investigating the broader PnecB group that included PnecB1 and PnecB2 (Wu and Hahn, 2006a, b; Newton et al., 2011; Jezbera et al., 2012), Relations of subcluster abundances with conductivity were similar to those with pH, as a corrolary of the positive correlation between the two physico-chemical parameters (Supplementary Figure S12).

The assignment of OTUs beyond the subcluster level yet suggests intra-subcluster differentiation and diversity that has only been demonstrated for PnecC (Hahn et al., 2016) and LimC (Jezbera et al., 2013; Kasalický et al., 2013) before. We found that OTUs within subclusters showed partly contrasting preferences, and even single OTUs exhibited broad ecological preferences along environmental gradients (Figure 4). These results suggest that ecological diversification is not only limited to the PnecC subcluster, but pronounced in other subclusters as well. Geography had the second largest impact on *Limnohabitans* and *Polynucleobacter* community composition. While a noticeable geographic pattern for *Polynucleobacter* resulted from a pronounced PnecA richness in Scandinavian lakes, we did not observe a geographic pattern for *Limnohabitans*. The investigated taxa were least affected by the composition of microeukaryotes. The weakness of the signal is probably caused by partially opposing effects of complex interactions between bacteria and microeukaryotes.

### Ecological Diversification

Among *Polynucleobacter*, PnecC is to date the best characterized subcluster considering diversity, ecophysiology, and genomic traits. However, it was demonstrated that the 16S rRNA gene does not reflect the diversity of PnecC yet rather conceals its taxon richness, which is revealed by extensive genomic differences of strains with similar 16S sequence (Hahn et al., 2016). The coarse resolution of the 16S rRNA gene and indications for a strong diversification in PnecC are also supported by our analyses on OTUs assigned to this subcluster. The phylogenetic analysis of the partial 16S rRNA sequences revealed that the OTU C_77 of the subcluster PnecC matches the sequences of two described PnecC species (Figure 1). Further, C_77 exhibited exceptionally broad ecological preferences, specifically in latitudinal distribution, conductivity, and pH (Figure 4). While a pan-European distribution was determined for *Polynucleobacter paneuropeaus* (Hoetzinger et al., 2019) that has a matching 16S rRNA gene sequence with C_77, the covered conductivity and pH ranges of C_77 likely exceed the niche this species might be adapted to. Six strains of *P. paneuropeaus* were isolated from habitats with conductivities and pH values ranging from 15–55 μS cm^–1^ and 4.6–7.5, respectively (Hoetzinger et al., 2019), which cover only a small part of the environmental range C_77 occured in. The type strain of *Polynucleobacter hirudinilacicola*, also comprised in the OTU C_77, was isolated from a pond with relatively high conductivity (353 μS cm^–1^) and a pH of 8.0 (Hahn et al., 2018). Possibly, this or other yet undescribed species may contribute to detections at higher conductivity and pH values of the OTU.

Although PnecC was considered an exception regarding ecological differentiation, we found indications for similar differentiation in two more *Polynucleobacter* subclusters. PnecB shows divergent ecological signals among the subclusters B2 and B1, and within B1 itself. Specifically, the subcluster B2 matched previously observed features of PnecB, i.e., positive correlations with pH and its absence from acidic habitats (Wu and Hahn, 2006b; Newton et al., 2011), while PnecB1 indicated a weak relation to physico-chemical factors except for latitude and temperature. The vague ecological signal of PnecB1 most probably results from OTUs assigned to this subcluster having different ecological preferences as indicated by different pH and conductivity ranges for the two assigned OTUs (Figure 4). While B1_367 preferentially occurs in lakes with alkaline pH and a measured conductivity between 100 and 1000 μS cm^–1^, B1_1350 occurred in circum-neutral lakes with low conductivity up to 200 μS cm^–1^. This observation suggests that subcluster B1 comprises at least two differentially adapted species. Thus, PnecB is composed of highly divergent taxa whose unique ecological adaptations are concealed by the coarse subcluster level.

Another example for ecological diversification was revealed in PnecA. Although PnecA was overall the rarest subcluster in the dataset, which corroborates estimations from previous studies (Hahn et al., 2011; Newton et al., 2011), it comprised significantly more OTUs than the other *Polynucleobacter* subclusters. These findings suggest that the PnecA subcluster comprises an exceptionally high 16S rRNA sequence diversity and significantly contributes to the *Polynucleobacter* diversity, in particular in Scandinavian samples (Figure 5). More sampling at these sites during different seasons and from more northern lakes would help to validate the high 16S rRNA sequence diversity of PnecA in this region. Despite the high number of PnecA OTUs, most of them exhibit similar occurrence patterns, suggesting a coherent ecology of most PnecA taxa compared to PnecC and PnecB. An exception was the most abundant OTU (A_647) occurring at a broader ecological range (Figure 4). Interestingly, this OTU shares sequence identity with the type strain of the only so far described PnecA species, *Polynucleobacter rarus* (Hahn et al., 2011), and was most abundant in lakes with alkaline pH between 8 and 9. In contrast, the type strain of *P. rarus* was isolated from the acidic Crystal Bog Lake (pH ∼ 4.5) located in North America (Newton et al., 2006). This may indicate ecological diversification within OTU A_647, similarly as described above for OTU C_77. Hence, ecologically distinct species with similar 16S rRNA might be also present in other *Polynucleobacter* subclusters than PnecC.

*Limnohabitans* has been shown to comprise bacteria with larger genomes (Kasalický et al., 2018), bigger cell sizes (Kasalický et al., 2013), and presumably higher overall growth rates (Simek et al., 2006) as compared to *Polynucleobacter* (Hahn et al., 2017). Thus, a more opportunistic lifestyle has been proposed for *Limnohabitans* bacteria, which might also be reflected in more generalist adaptations (Kasalický et al., 2013). Nevertheless, extensive phenotypic and ecological diversification has been demonstrated within the subcluster LimC as well (Jezbera et al., 2013; Kasalický et al., 2013), which might be comparable to the diversification within PnecC discussed above. A difference to *Polynucleobacter* is a higher diversity of *Limnohabitans* on 16S rRNA sequence level, as evident from 51 OTUs assigned to *Limnohabitans* subclusters opposed to 25 *Polynucleobacter* OTUs (Figures 1, 2). Most OTUs were assigned to LimC (27 OTUs) and LimB (14 OTUs). Within LimC, for instance OTUs C_605 and C_961 show opposing preferences regarding pH and conductivity. The former is more abundant at alkaline pH (8.5–9.2) and high conductivities, while the latter was preferentially detected at lower pH (7.5–8.1) and low conductivities.

Also within certain *Limnohabitans* OTUs, broad environmental preferences may indicate cryptic diversity rather than generalist adaptations. For example, OTU A_329 was abundant in the most acidic lake sampled (Lake Černé Jezero, pH 5.4), and rare or absent in circum-neutral lakes, but showed relatively high abundances in lakes with pH > 7.5. Such a distribution pattern would be unlikely for a single generalist phenotype.

Especially interesting is the most abundant *Limnohabitans* OTU D_147, identical in partial 16S sequence with Uncultured clone PIB-25 (Figure 2). This OTU shows surprisingly similar distribution patterns to the *Polynucleobacter* OTU C_77, which comprises at least two different species (see above). Unfortunately, no isolates affiliated to LimD are available to date, and it can thus only be speculated if this subcluster comprises a species cluster comparable to PnecC. In summary, our study indicates extensive ecological diversification within all *Polynucleobacter* and *Limnohabitans* subclusters. Cultivation as well as cultivation-independent studies with sequences providing higher phylogenetic resolution would be necessary to better characterize this diversification.

### Diversity and Geography

The magnitude and large geographic range of sampling sites allowed us to investigate *Polynucleobacter* and *Limnohabitans* diversity, i.e., OTU richness and diversity indices, in light of biogeography. In this context, we focused on the latitudinal diversity gradient and the species-area relationship. Briefly, the latitudinal diversity gradient suggests that species richness declines from the tropics to the poles (Hillebrand, 2004). The species-area relationship assumes a positive correlation between ecosystem size and observed number of species (MacArthur and Wilson, 1967). While Reche et al. (2005) found a positive species-area relationship for bacterial communities using 16S rRNA OTUs as diversity unit, we observed no significant correlation of lake area with OTU richness in our study, neither for *Polynucleobacter* nor for *Limnohabitans* (Figure 5 and Supplementary Figures S9, S10). Yet, Shannon and Simpson index of *Limnohabitans* showed a weak positive correlation with lake area (Supplementary Figure S9). Regarding the latitudinal diversity gradient hypothesis, *Limnohabitans* diversity indices did not significantly correlate with temperature and latitude. However, *Polynucleobacter* diversity indices were negatively correlated with temperature and positively with latitude. This contradicts the assumptions of the latitudinal diversity gradient hypothesis. However, diversity did not increase gradually with latitude but the positive correlation was mainly caused by a particularly high diversity in the most northern region of our sampling area (Figure 5).

We suppose two underlying causes for this geographic pattern. Firstly, the regional characteristics in the sampled Scandinavian area may promote *Polynucleobacter* abundance (Supplementary Figure S8), and consequently its diversity. Logue and Lindström (2008) state the importance of regional factors for the diversity and biogeography of bacterioplankton in inland waters. In their concept, lakes are regarded as parts of larger units (i.e., their drainage areas), and thus the region the lakes are located in affects their local physico-chemical characteristics (e.g., pH). As these characteristics in turn affect the local bacterial communities, they are referred to as indirect regional effects on community composition. In addition, the import of bacteria from the regional metacommunity poses direct effects on the local communities (Logue and Lindström, 2008). The importance of such dispersal effects is exemplified by the island biogeography theory (Connor and McCoy, 1979), if lakes are viewed as islands within a terrestrial landscape (Dodson, 1992). Due to a high density and connectivity of lakes in Scandinavian areas, both indirect and direct regional influences might be particularly important for resident bacterial communities. Accordingly, Logares et al. (2013) observed similar physico-chemical characteristics for different lakes from a region in Scandinavia, and estimated high dispersal among these lakes. The Scandinavian lakes in our study are also characterized by similar environmental features, i.e., low pH and temperature (Supplementary Table S1), which favor the abundance of PnecC and PnecA (Figure 3 and Supplementary Figures S5, S8). Large population sizes and high dispersal rates may consequently account for high *Polynucleobacter* diversities within single lakes.

A second factor accounting for the high OTU richness observed in this area is the specifically high contribution of PnecA to the regional metacommunity. PnecA is characterized by a high 16S rRNA gene diversity compared to the other *Polynucleobacter* subclusters (Figure 1). Thus, the OTU richness in this region may be disproportionally high due to the used marker and does not necessarily represent an equally higher species richness.

Although we could not find a latitudinal gradient and no clear species-area relationship by analyzing 16S rRNA gene amplicons, we cannot exclude that such patterns would become evident when using phylogenetic markers with higher resolution. The 16S rRNA gene in this respect suffers from limitations to resolve species diversity, as discussed above. According to calibrated molecular clocks from endosymbiotic bacteria, the maximum evolutionary rate of the 16S rRNA gene is 0.091% sequence divergence per million year (Kuo and Ochman, 2009). This suggests that possible patterns caused by recent temporal events (e.g., the last glacial period c. 115 000 – c. 11 700 years ago) cannot be detected with this marker.

### Microbial Interactions of Microeukaryotes and Both Taxa

The effect of eukaryotes on the composition of *Polynucleobacter* and *Limnohabitans* was the lowest in comparison to geography and physico-chemical factors (Table 1). The overall low correlation coefficients might suggest varying trophic interactions of eukaryotes and bacteria among sites (Figure 6). Remarkably, *Limnohabitans* subcluster show an overall stronger interconnection in the network compared to *Polynucleobacter* suggesting members of *Limnohabitans* being highly interactive. In this respect, LimB seems to play a major role in the network. For example, LimB shows a comparably strong connection to the groups of eukaryotic flagellates Cryptophyta, Choanoflagellida, and Chrysophyta. These flagellates increased in relative abundance in grazing experiments with LimB (Grujcic et al., 2018). Interconnections with these and other eukaryotes may also harbor different qualities of interactions besides predator-prey relationships, such as utilization of eukaryotic exudates by bacteria (Simek et al., 2011; Horňák et al., 2017). For PnecB, which shows the strongest interconnection of the *Polynucleobacter* subclusters in our network, it was demonstrated that they grew on organic matter derived by chlorophyte taxa, i.e., *Coelastrum reticulatum* (Horňák et al., 2017). Our results do not show a connection of PnecB2 and Chlorophyta but with other eukaryotic groups that comprise exudate releasing taxa. It is possible that PnecB2 species specifically profit from exudates of other, so far not tested eukaryotes. Additionally, seasonal effects and grazing pressure by zooplankton, as demonstrated by Jezberová et al. (2017), affect the abundance of microeukaryotes and therefore the correlations with bacteria. Summarized, the analyzed co-occurrences might indicate direct interrelations, however, we cannot exclude the presence of unobserved interactions. For instance, interactions among bacteria and eukaryotes with opposing effects on bacterial abundance, i.e., grazing and exudate utilization, may mitigate signals of interrelation. Diverging exudate preferences of different taxa within bacterial subclusters may also contribute to the weakening of correlations.

## Conclusion

Our study emphasizes the importance of physico-chemical factors on the distribution of bacterial taxa affiliated to *Polynucleobacter* and *Limnohabitans* in comparison to composition of microeukaryotes and geographical ecosystem properties. Along those physico-chemical gradients, we could demonstrate that the ecological diversification within PnecC and LimC is not an exception within the genera *Polynucleobacter* and *Limnohabitans*, but likely applicable for the other subclusters as well. We identified geographic patterns in OTU richness and abundance for both taxa that indicate the importance of region-specific environmental conditions for local community composition. A relationship between area or latitudinal gradient and taxon richness could not be identified, however, due to the limited phylogenetic resolution of the 16SrRNA gene we cannot exclude the presence of such relationships. Finally, observed co-occurrence patterns between bacterial and microeukaryotic taxa provide a basis for future research to test for specific bacteria-protist interactions in freshwater ecosystems.

## Data Availability Statement

The datasets generated for this study can be found in the NCBI Sequence Read Archive BioProjects PRJNA559862 and PRJNA414052.

## Author Contributions

All authors contributed to the study design, read, and approved the final version of the manuscript. JN analyzed the sequencing data and performed the statistical analyses. MH conducted the phylogenetic analyses. JN and MH wrote the manuscript. JB, MWH, and DB contributed to the final stages of writing.

## Conflict of Interest

The authors declare that the research was conducted in the absence of any commercial or financial relationships that could be construed as a potential conflict of interest.

## Footnotes

1 https://github.com/MW55/Natrix
